# Psychological correlates of body dissatisfaction in Swiss youth over a one-year study-period

**DOI:** 10.3389/fpsyg.2023.1269364

**Published:** 2024-01-08

**Authors:** Verena M. Mueller, Felicitas Forrer, Andrea H. Meyer, Simone Munsch

**Affiliations:** ^1^Department of Psychology, Clinical Psychology and Psychotherapy, University of Fribourg, Fribourg, Switzerland; ^2^Department of Clinical Psychology and Epidemiology, Institute of Psychology, University of Basel, Basel, Switzerland

**Keywords:** body dissatisfaction, muscle dysmorphic symptoms, adolescence, young adulthood, emotion regulation difficulties, alexithymia, appearance-based rejection sensitivity

## Abstract

**Introduction:**

It is well known that young individuals often report pronounced negative perceptions and attitudes towards their own body or intense fear of being not muscular enough. There is much less data available, however, on the role of psychological mechanisms on these perceptions and attitudes, such as emotion regulation difficulties, correlates of alexithymia, and appearance-related rejection sensitivity.

**Methods:**

We therefore set out to assess associations between these psychological mechanisms, and body image as well as muscle dysmorphic symptoms. Our sample was recruited as part of a larger-scale study aiming at assessing correlates of mental health (with a focus on eating disorder symptoms) in German speaking Switzerland. The first wave (T1), starting in April 2021, included 605 participants (80% female, 19.6 ± 2.5 years) who completed the online-questionnaire and were reassessed in a second wave (T2), one year later.

**Results:**

Results indicated that at both waves, emotion regulation difficulties [DERS-SF] and appearance-based rejection sensitivity [ARS-D] were both positively cross-sectionally associated with body dissatisfaction [BSQ-8C] and muscle dysmorphic symptoms [MDDI] at the first assessment time-point and one year later at follow-up assessment. Moreover, alexithymia [TAS-20] was positively cross-sectionally associated with muscle dysmorphic symptoms at both waves. We further observed high absolute and relative level stabilities for all variables involved across the one-year study period.

**Discussion/Conclusion:**

Even though the effects for some associations were rather small, our findings underline the relevance of such mechanisms in the development of body dissatisfaction and to a lesser extent of muscle dysmorphia symptoms over the period of one year. Additional research is necessary to replicate these findings in other youth samples.

## Introduction

Body dissatisfaction (BD) is an important element of body image which encompasses the negative self-evaluation of physical appearance and consequently influences how people think, feel, perceive, and act with regard to their bodies (Thompson and Cafri, 2007; Schuck et al., 2018). In this context, BD is based on cognitive and emotional aspects and can vary under the influence of psychological (e.g., emotion regulation difficulties), biological (e.g., body mass index) or sociocultural factors (e.g., educational status; Blake, 2015).

Research from Western society populations indicates that BD is a prevalent condition and is often referred to as normative discontent, which can occur throughout the lifespan (Tantleff-Dunn et al., 2011). Especially adolescence may be characterized as a period of particular vulnerability in the development of BD, with major physical, social, cognitive and emotional changes, and identity formation occurring (Rickwood, 2015; Voelker et al., 2015). Many adolescents place significant importance on their appearance, as attractiveness often brings higher social status in their peer groups (Borch et al., 2011; Ricciardelli and Yager, 2015), and are therefore more vulnerable to the development of BD (Grosick et al., 2013). Also, young adulthood represents a distinctive developmental stage characterized by significant transitions (e.g., increasing autonomy), self-identity formations and physical changes (e.g., weight gain, changes in body proportion; Bucchianeri et al., 2013; Wang et al., 2019). Given that, high BD may hinder a healthy transition from adolescence into adulthood and relates to the development of mental health impairment (McLean et al., 2022). In a recent report published by Health Promotion Switzerland, it was found that only 35% of females and 56% of males aged 13–17 (*n* = 371; 47.4% females) expressed satisfaction with their appearance (Widmer et al., 2018). These prevalence rates of BD in Swiss youth are comparable with previous research conducted in Europe, Australia and the North America (Frisén et al., 2015; Aanesen et al., 2017; Hoffmann and Warschburger, 2018; Wang et al., 2019; Lacroix et al., 2022; McLean et al., 2022). To the best of the authors knowledge, no research is currently available regarding the prevalence of BD among young adult samples in the German-speaking region, highlighting the need of new studies within this age group.

Over the last decade, BD has been recognized as a serious public health concern (McLean et al., 2022), with onset typically occurring in early adolescence (Rohde et al., 2015). For instance, BD has been identified as one of the most important and growing risk factors in the development and maintenance of eating disorders (Rohde et al., 2015; Stice et al., 2017; Sharpe et al., 2018a) and has also been shown to occur in individuals with depressive (Brechan and Kvalem, 2015) and social anxiety disorder (Bijsterbosch et al., 2020) or body dysmorphia (Arji et al., 2016), as well as in individuals without mental disorders (Bucchianeri et al., 2013; Mond et al., 2013; Frisén et al., 2015). Moreover, BD is associated to low self-esteem (Wojtowicz and von Ranson, 2012), overweight/obesity (Loth et al., 2015), emotional and negative mood distress (Ferreiro et al., 2014; Sharpe et al., 2018b) and reduced overall quality of life (Griffiths et al., 2016). BD further predicts unhealthy behaviors, such as strategies to reduce weight (e.g., restrictive dieting, excessive exercise) and increase muscularity (e.g., extreme weightlifting, steroid misuse), in order to conform a certain thin- or muscular-ideal (McLean et al., 2022).

Particularly in more recent research muscle dysmorphia (MD) has gained interest (Edwards et al., 2016; Klimek et al., 2018; Mitchison et al., 2022). MD is defined by a preoccupation around muscularity as well as a distorted perception of one’s own body (Pope et al., 1997; McFarland and Kaminski, 2009) and was included as a specifier of body dysmorphic disorder in the fifth edition of the Diagnostic and Statistical Manual for Mental Disorders (DSM-5, American Psychiatric Association, 2013). Individuals with MD engage in unhealthy body change behaviors with the goal of achieving muscular hypertrophy and low body fat levels (mesomorphic ideal). This may include extreme exercise, strict dieting to either gain weight (for muscularity; e.g., high-protein intake) or to lose weight (for thinness and low body fat; e.g., fasting) and the use of anabolic steroids or other muscle-enhancing substances (e.g., protein powder; Klimek et al., 2018; Nagata et al., 2020). Despite having a well-developed muscular physique, individuals with MD frequently perceive themselves as lacking in leanness and muscularity, resulting in elevated levels of BD (e.g., avoidance of body exposure; Schuck et al., 2018; Mitchison et al., 2022).

However, currently little is known about mechanisms related to MD as the most frequent used BD questionnaires primarily assess weight and shape concerns but do not capture dissatisfaction with and drive for muscularity. This is why the frequency of BD, especially among young males, has been potentially underestimated so far. While MD is predominantly observed in males (Pope et al., 1997; Phillips, 2011), recent studies indicate a potential increase in the drive for muscularity and related behaviors among females. However, the research of MD has mainly focused on individuals seeking treatment or those involved in bodybuilding and competitive sport (e.g., González-Martí et al., 2014), rather than being conducted in the general youth population. As a result, there is a notable absence of epidemiological data concerning the prevalence of MD among young individuals, despite the likelihood of compulsive muscularity-related behaviors being most prevalent during late adolescence (Nagata et al., 2020). Given that, in the current study we extend the concept of BD by including symptoms of MD (from now on included in the abbreviation BD) and aim to provide additional information on the development of BD during youth.

Considering the well-known negative impact of BD, it is crucial to understand the mechanisms that contribute to its development and persistence. Research suggests that the perception and satisfaction of youth with their body shape, body size and muscularity are influenced by a variety of bio-psycho-social factors (Cafri et al., 2005; Rodgers et al., 2020). It is well known that BD is affected by *biological* factors such as age, gender and body weight (e.g., Wyssen et al., 2016; Nelson et al., 2018; Vuong et al., 2021; Frederick et al., 2022) as well as *social* factors including appearance pressure from peers and families or media (e.g., Castellanos Silva and Steins, 2023) as well as the role of socio-educational status (e.g., Ramos et al., 2019).

Body weight, or *body mass index (BMI)*, probably has the closest link to BD during youth. Independent of gender, higher BMI has been strongly associated with increased BD in various cross-sectional and longitudinal studies (e.g., Holsen et al., 2012; Sarrar et al., 2020; Frederick et al., 2022), including research conducted among Swiss adolescents (Knauss et al., 2008). Furthermore, increasing BMI over time was associated with higher BD in adolescence and early adulthood, whereas individuals who transitioned to a lower BMI category reported greater satisfaction with their bodies (Eisenberg et al., 2006). Due to the consistency of these findings regarding the importance of weight in the development of BD, we chose to control for BMI in the present study and concentrated on the role of psychological factors and their respective impact at two time points, one year apart.

Within the scope of *psychological* factors, emotion regulation difficulties (ERD) are likely to be of a high potential to influence BD. Emotion regulation is the capacity of young individuals to identify, recognize, express and regulate short and longer lasting affective and emotional states and refraining from impulsive behavior. ERD are especially prevalent in youth, where emotion regulation abilities are still developing (Riediger and Luong, 2015). ERD refer to both cognitive as well as behavioral obstacles related to identifying emotions and modulating emotional responses (Thompson, 1994). According to Gratz and Roemer (2004) this includes six distinct but related dimensions: lack of emotional clarity and awareness, difficulties engaging in goal-directed behavior, non-acceptance of emotional responses, impulse control difficulties and limited access to emotion regulation strategies. These dimensions have direct connections to other emotion-related factors, such as alexithymia, facilitating meaningful comparisons (Trompeter et al., 2021). In the present study, we have therefore chosen to additionally assess alexithymia, which refers to the inability to identify, understand, and express one’s own emotions, as well as a limited capacity to recognize and interpret emotions in others (Sifneos, 1973). The relevance of ERD in the maintenance of BD has been confirmed in a recent cross-sectional study involving Canadian adolescent males (*n* = 423, 14.7 ± 1.1 years). The authors found that higher levels of ERD were associated with increased levels of BD, engagement in muscle-building behaviors, and restrained eating (Morin and Meilleur, 2023). In a US American sample of undergraduate males (*n* = 196, 18.9 ± 1.4 years), ERD were identified as a significant predictor of BD, even after controlling for BMI and negative affect (Lavender and Anderson, 2009). Data from 551 Italian males (20.82 ± 4.43 years) demonstrates that ERD amplify the association between BD and the desire for muscularity (Dakanalis et al., 2015). Moreover, Swedish students (*n* = 1,265, 54.5% female, 13.5–19 years) with exceedingly high BD scores reported higher ERD (Hansson et al., 2016). Also, a study conducted with Australian high school and university students (*n* = 756, 60% female, 17.94 ± 1.98 years) found that increased levels of ERD were associated with higher reports of body dysmorphic symptoms (Gardner et al., 2021). Sim and Zeman (2005) discovered ERD to be significant predictors of BD and disordered eating in US American adolescent females (*n* = 235, mean age: 12.11 years), even after accounting for age and BMI. A study conducted by Hughes and Gullone (2011) among Australian adolescents (*n* = 533, 61% female, 15.6 ± 2.5 years) found that a greater reliance on dysfunctional ER strategies was associated with more BD, restrained eating, and bulimic tendencies. Moreover, 18-29-year-old students (*n* = 603, 77.6% female) with higher ERD showed elevated levels of BD and eating disorder symptoms, as well as decreased self-esteem. Conversely, emotion regulation strategies employed to deal with BD have been associated with psychosocial functioning (Cash et al., 2005). Findings of an Australian study (Cunningham et al., 2020) suggest that females (*n* = 221, 20.0 ± 3.55 years) with ERD, experience a higher drive for muscularity attitudes and behaviors over and above BMI and general negative affect. Furthermore, males (*n* = 304, 22.49 ± 4.38 years) with higher levels of alexithymia were more likely to report MD symptoms (Leone et al., 2015).

Besides ERD, young individuals are particularly responsive to signs of rejection from others, especially their peers (Webb et al., 2017). Appearance-based rejection sensitivity (ARS) is a relatively stable personality construct and has been described as the tendency to anxiously expect, readily perceive, and overreact to signs of rejection based on personal appearance (Park, 2007; Park and Pinkus, 2009). ARS consists of an affective and a cognitive component, whereas anxious concerns of appearance rejection represent the affective component and expectations of appearance rejection the cognitive component (Park, 2007). ARS has been associated with a number of psychological impairments, including low self-esteem, social anxiety, depressive symptoms, disordered eating and peer victimization (e.g., Park, 2007; Bowker et al., 2013; Webb and Zimmer-Gembeck, 2015; Roberts et al., 2018; Hawes et al., 2020). Moreover, individuals who experience high levels of ARS tend to interpret even ambiguous signs of rejection as confirmation of their fears. This leads them to withdraw from social interactions, which in turn contributes to their social isolation and fear of rejection (self-defeating prophecy; Park and Pinkus, 2009; Bowker et al., 2013). In cross-sectional studies involving adolescents and young adults, it has been found that females tend to report higher levels of ARS compared to males (Park et al., 2009; Webb et al., 2014; Webb and Zimmer-Gembeck, 2015) and that higher ARS predicted BD, acceptance of cosmetic surgery, and disordered eating (Park et al., 2009). A current study among Australian adolescents (*n* = 897, 42.21% female, 14.8 ± 1.4 years) examined the moderating role of ARS in the relationship between appearance-based peer victimization and increased weight/shape concerns in a one-year follow-up (Trompeter et al., 2022). Results illustrated an association between current appearance-related victimization and weight/shape concerns, which was dependent on ARS. However, stronger associations were found for lower levels of ARS. This study’s results are the only findings contradicting earlier research from different countries that proposed adolescents with a heightened ARS may be more vulnerable to weight/shape concerns when experiencing peer victimization (Lavell et al., 2014; Schmidt and Martin, 2019). In a sample of British university students (*n* = 106, 77.4% female, 21.54 ± 8.34 years) higher levels of ARS were further positively associated with more excessive BD (e.g., “how worried are you about the way that you look?”) and with acceptance of cosmetic surgery (Calogero et al., 2010). Moreover, Australian high school and university students (*n* = 756, 60% female, 17.94 ± 1.98 years) reported more body dysmorphic symptoms when they experienced more appearance-based rejection by their peers (Gardner et al., 2021).

Although previous research shows that many adolescents and young adults do have very high concerns about appearance rejection, no studies have examined the influence of ARS on BD in a general youth sample cross-sectional and one year later. So far, most studies have predominantly focused on university students or patients with body dysmorphic disorder and were conducted cross-sectionally. Moreover, ARS is poorly researched in the German-speaking region owing to a lack of assessment instruments. Beyond that, the influence of ARS has been primarily examined on body dysmorphic concerns, but not on BD (e.g., weight, shape, and muscularity concerns).

In sum, despite the findings from existing cross-sectional and longitudinal studies that have identified *biological*, *social*, and *psychological* factors associated with BD, there continues to be a lack of data on psychological mechanisms such as struggles with regulating negative emotions and concerns with appearance contributing to the development of both BD and symptoms of MD cross-sectionally over time. In order to improve prevention and treatment interventions targeting BD, it seems crucial to enhance our understanding of the punctual and/or longer lasting influence of ERD, alexithymia and ARS in a youth sample from the general population. Therefore, the goal of this study was to determine the influence of ERD, alexithymia and ARS on BD and MD cross-sectionally, at two different time points, one year apart. We hypothesized that higher values of impairment in emotion regulation, alexithymia and hastily perception of appearance-related rejection are associated with higher measures of BD and MD symptoms, while controlling for age, gender, BMI and educational status in our Swiss youth sample at both time points.

To better understand how the investigated variables (correlates and outcomes) develop over a one-year period, we assessed absolute and relative level stability. Absolute level stability deals with average temporal change, i.e., whether mean values of a characteristic change or remain on the same level over a one-year study period. Relative level stability, in contrast, deals with individual temporal change, i.e., whether individuals differ in their temporal change over the one-year time period from each other and thus whether the correlation between values of a characteristic measured one year apart is high or low (serial correlation; Newsom, 2015).

## Materials and methods

### Study design

This longitudinal online-questionnaire study is part of a large-scale, Swiss research project (Binge-Eating Adolescent and Young Adults Treatment; i-BEAT) conducted at the University of Fribourg (Switzerland). It was started in April 2021 with the overarching aim to assess determinants of mental health – especially disordered eating behaviors such as loss of control eating – in a general youth population. In the online-questionnaire sub-study presented here, we assessed mental health correlates of a German-speaking general youth population across two measurement points, one year apart (baseline, 12-month follow-up), using the platform Qualtrics. The study received approval from the cantonal ethical committee of Bern, has been registered (Study ID: 2019–01277; DRKS-ID: DRKS00023706) and the protocol has been published (Munsch et al., 2021a,b). The present report is the first to report results from the i-BEAT study.

### Participants

Between April 2021 and May 2023, a total of *n* = 605 adolescents and young adults participated. Inclusion criteria for study participation in this online-questionnaire study were age (14–24 years), sufficient German language proficiency, absence of pregnancy or lactation and online written informed consent. Recruitment was conducted via social media (Instagram, LinkedIn), mailing lists at local schools and universities, flyers, newspapers and mental health institutions in Switzerland and Germany. For the 12-month follow-up, participants received 20 Swiss francs, whereas the screening was not financially compensated. Psychology students from the University of Fribourg (Switzerland) received course credits for participation.

### Procedure

Registration of participants was conducted through our study-website. Subsequently, participants were emailed a link to the online-questionnaire and first provided with the information and aims of the study, to which they had to give online informed consent in order to proceed. The questionnaire then started with an assessment of the sociodemographic characteristics, followed by different mental health questionnaires (for the selection used in this study see measures and a detailed overview see Munsch et al., 2021a,b). Completion of the whole online-questionnaire took approximately 30–45 min and was anonymous. A progress bar provided an overview on the percentage of completed questions. The e-mail address was used to create a unique 64-digit ID, thereby allowing the online-questionnaires from the two measurement points to be matched. For the encryption process the SHA-256 algorithm was used.

### Assessment instruments

#### Sociodemographic data

In order to assess sociodemographic characteristics of the sample, the online-questionnaire included self-reported items on participant’s gender (male, female, other), current age (in years), educational status, current body weight (in kg) and height (in cm). Body mass index (BMI) was calculated using the standard formula (kg/m^2^; Weir and Jan, 2023).

#### Correlates of general mental health in a youth population

The Patient Health Questionnaire-4 (PHQ-4; original version: Kroenke et al., 2009; German version: Löwe et al., 2010) is an ultra-brief self-report questionnaire, which combines the 2-item depression scale (PHQ-2) and the 2-item anxiety scale (GAD-2), each provided on a 4-point Likert scale (0 = not at all; 3 = nearly every day). The PHQ-4 has been widely used in research and clinical settings, within a range of populations. Moreover, the PHQ-4 shows good construct validity, with associations between the PHQ-4 and scales from the Medical Outcomes Study Short-Form General Health Survey (SF-20; Kroenke et al., 2009). The strongest association was observed between the PHQ-4 and mental health (0.80). For the present study, we therefore decided to use the PHQ-4 total score as a comprehensive measure of general mental health (see Table 1). The PHQ-4 total score was computed as a sum score, ranging from 0 to 12, with higher scores indicating greater mental health impairment (0–2 = normal; 3–5 = light, 6–9 = moderate; ≥ 10 = severe). Among others, the PHQ-4 correlates well with age, gender and education level, which are established risk factors for anxiety and depression (Löwe et al., 2010). Cronbach’s alpha and McDonald’s omega in the present sample were 0.82 and 0.85, respectively. Just recently Wicke et al. (2022) provided an update of normative data from the German general population and suggested that scores of six or higher prompt a more detailed clinical interview for diagnosis of depression or anxiety disorders.

**Table 1:** Sample’s characteristics with respect to measures at T1 and T2.

Variable	T1 (screening)	T2 (follow-up)
*n*	605	260
	*n* (%)	*n* (%)
Gender		
Female	481 (80)	219 (84)
Male	112 (19)	37 (14)
Non-binary	12 (2)	4 (2)
COVID-19		
During restrictions	217 (36)	0 (0)
After restrictions	388 (64)	260 (100)
Nationality		
Swiss	412 (68)	181 (70)
German	150 (25)	78 (30)
Other	43 (7)	1 (<1)
Education		
Still in school	278 (46)	118 (45)
At university	289 (48)	131 (50)
In training	8 (1.3)	1 (0.4)
Employed	16 (2.6)	4 (1.5)
Other	14 (2.3)	6 (2.3)
	M (SD)	M (SD)
Age (years)	19.6 (2.5)	20.3 (2.5)
Body Mass Index (kg/m^2^)	22.1 (3.5)	21.9 (3.6)
BSQ (total score)	23.4 (11.1)	20.8 (10.0)
MDDI (total score)	10.6 (4.2)	10.5 (4.0)
ARS-D (total score)	10.6 (4.5)	10.1 (4.2)
DERS-SF (total score)	40.3 (12.4)	38.1 (12.0)
TAS-20 (total score)	49.6 (11.9)	48.4 (12.3)
PHQ-4 (total score)	7.8 (2.6)	7.5 (2.6)
EDE-Q8 (total score)	2.0 (1.7)	1.6 (1.4)

BSQ, Body Shape Questionnaire (short form); MDDI, Muscle Dysmorphic Disorder Inventory; ARS-D, Appearance-related Rejection Sensitivity; DERS-SF, Difficulties in Emotion Regulation Scale (short form); TAS-20, Toronto-Alexithymie-Skala-20; PHQ-4, Patient Health Questionnaire for anxiety and depression; EDE-Q8, Eating Disorder Questionnaire (short form); percentages do not always add up to 100% due to rounding.

Global eating disorder psychopathology was assessed using the total score of the 8-item short version of the Eating Disorder Examination (EDE-Q8; original German version: Kliem et al., 2016). The EDE-Q8 consists of two items from each of the four subscales: eating concern, weight concern, shape concern and restraint during the past 28 days, rated on a 7-point Likert scale (0 = no day, 6 = every day). The total score (range: 0–6) is calculated by summing the means of the subscales and dividing the obtained value by the number of subscales, with a higher total score indicating higher eating disorder psychopathology. According to Kliem et al. (2016), the EDE-Q8 is particularly suitable for use in epidemiological research, when an economical assessment of global eating disorder psychopathology is required. Based on good psychometric properties, including an excellent correlation (r = 0.97) with the original and well-established 36-item Eating Disorder Examination-Questionnaire (EDE-Q; original version: Fairburn and Cooper, 1993; German version: Hilbert and Tuschen-Caffier, 2016) the use of the EDE-Q8 appears to be appropriate. Cronbach’s alpha and McDonald’s omega in the present sample were 0.95 and 0.97.

#### Outcomes

Currently, there is no questionnaire on BD available, which includes also symptoms of MD. The construct, BD, is therefore assessed using the following two validated questionnaires:

We used the total sum score of the German short-version of the Body Shape Questionnaire (BSQ-8C; original version: Evans and Dolan, 1993; German version: Pook et al., 2002) to quantify BD within the last four weeks prior to the two assessments (T1 and T2). It contains 8 gender non-specific items, which are scored on a 6-point Likert scale (1 = never; 5 = always) with higher scores indicating greater trait BD (range: 8–40). Values below 19 indicate no concerns with body shape, scores from 19–25 mild concerns, and values above 25 moderate to marked concerns (Evans and Dolan, 1993). Two items of the BSQ-8C ask for feelings about being fat or worries about becoming fat. The other items include situational and personal causes, as well as consequences of feeling fat. The BSQ-8C has high internal consistency, excellent test–retest reliability as well as high convergent validity and is therefore very well suited to investigate the occurrence and change in BD (Pook et al., 2008; Welch et al., 2012). Cronbach’s alpha and McDonald’s omega in the present sample were 0.94 and 0.95, respectively.

The Muscle Dysmorphic Disorder Inventory (MDDI; original version: Hildebrandt et al., 2004; German version: Zeeck et al., 2018) is a 13-item measure that assesses aspect of MD on three different subscales: drive for size (DFS; e.g., thoughts of being thinner), appearance intolerance (AI; e.g., negative beliefs about one’s body), and functional impairment (FI; e.g., excessive and compulsive exercise). Due to the increasing importance of muscularity (e.g., mesomorphic body) in both genders (Rodgers et al., 2018; Vuong et al., 2021; Mitchison et al., 2022), we decided to use the MDDI-total sum score, which can be derived from sum of the underlying subscales. The three-factor structure of the MDDI has been reported to be independent of gender with no statistically significant difference in the total sum score (range: 13–65; Zeeck et al., 2018). Participants rate statements on a 5-point Likert scale (1 = never; 5 = always), with higher scores indicating higher MD-related symptoms. The MDDI showed good internal consistency (total score: *α* = 0.75; Zeeck et al., 2018). Cronbach’s alpha and McDonald’s omega in the present sample were 0.80 and 0.88, respectively.

#### Psychological correlates of body dissatisfaction and muscle dysmorphic symptoms

The short-form of the Appearance-Based Rejection Sensitivity Scale (ARS-D; original version: Park, 2007; German version: Schmidt and Martin, 2017) assesses the degree to which individuals are anxiously concerned and expect to be rejected based on their appearance. The ARS-D originally contains 15 items that describe brief scenarios related to one’s appearance in different social contexts. For the present study, we used only four of these scenarios (items 1, 2, 4, 11) and adapted the language to our younger target population. Participants rated each scenario on a 6-point Likert scale to indicate their worries about being rejected (affective component; 1 = very unconcerned; 6 = very concerned) and the estimated likelihood of rejection (cognitive component; 1 = very unlikely; 6 = very likely) based on appearance. The ARS-D shows high internal consistency as well as convergent and discriminant validity (Park, 2007; Schmidt and Martin, 2017). For each scenario the scores of the affective and cognitive component are multiplied and averaged, leading to a total sum score (range: 1–36), where higher scores indicate stronger ARS. Cronbach’s alpha and McDonald’s omega in the present sample were 0.64 and 0.68, respectively and therefore lower than those reported in literature (*α* = 0.90 for the ARS global score, Park, 2007; *α* = 0.90 for the ARS-D global score, Schmidt and Martin, 2017).

ERD were examined by the short-form of the Difficulties in Emotion Regulation Scale (DERS-SF; original version: Kaufman et al., 2016). For the German translation we have followed the original items of the well validated and frequently used Difficulties in Emotion Regulation Scale (DERS; Gratz and Roemer, 2004) and used the same items for each shortened subscale as Kaufman et al. (2016). The DERS-SF consists of 18 items answered on a 5-point Likert scale (1 = almost never; 5 = almost always). A total of six subscales can be formed from three items each: lack of emotional clarity (*clarity*), lack of emotional awareness (*awareness*), difficulties engaging in goal-directed behavior (*goals*), non-acceptance of emotional responses (*non-acceptance*), impulse control difficulties (*impulse*) and limited access to emotion regulation strategies (*strategies*). Higher mean values in each scale (range: 1–5) represent greater difficulties in this facet of emotion regulation. The DERS-SF total score, which is calculated as total sum score (range: 18–90), was used to multidimensionally assess overall ERD. Cronbach’s alpha and McDonald’s omega in the present sample were 0.90 and 0.93, respectively. Kaufman et al. (2016) observed for the DERS-SF scales satisfactory to good reliability coefficients as well as indications of validity.

The Toronto Alexithymia Scale-20 (TAS-20; original version: Bagby et al., 1994; German version: Bach et al., 1996) is a 20-item self-report measure to assess the three components of alexithymia: difficulty identifying emotions (DIF), difficulty describing emotions (DDF) and an externally-orientated thinking style (EOT) whereby individuals rarely pay attention to their own emotions. All items can be rated on a 5-point Likert scale (1 = strongly disagree; 5 = strongly agree) and summed into a total score (range: 20–100) which we used as an overall marker of alexithymia. Five items are reversed scored (items: 3, 4, 10, 17, 18). Cronbach’s alpha and McDonald’s omega in the present sample were 0.86 and 0.88, respectively.

#### Covariates of body dissatisfaction

We further recorded the following variables which were used as covariates in our analyses: participants’ age (in years), gender (male, female, other), educational status, BMI and whether the assessment of the online-questionnaires took place during the COVID-19 pandemic or thereafter. We decided to choose April 1st 2022 as the cut-off date, as all remaining COVID-19 restrictions were lifted in Switzerland at this date (e.g., compulsory isolation of infected persons, compulsory use of masks in public; Federal Council of Switzerland, 2022).

#### Statistical analyses

Statistical analyses were performed using R, version 4.2.2. Correlates of outcomes (body dissatisfaction [BD], aspect of muscle dysmorphia [MD]) at both waves were analyzed using multiple linear regression models. Each model contained the respective outcome at T1 or T2, the three correlates (appearance-based rejection sensitivity [ARS], emotion regulation difficulties [ERD], and components of alexithymia), all measured at the same wave as the corresponding outcome, plus the covariates age, gender and educational status of the respondent, plus an additional covariate, denoting whether the assessment took place during the COVID-19 pandemic (i.e., before 1 April 2022) or thereafter. Note that at time of publication, data assessment for T1 was complete according to the study protocol of Munsch et al. (2021a,b), while data of the second assessment time point at T2 was not yet available for all participants. Thus, sample size at T1 were considerably higher than that for T2 (see Table 2) and we therefore analyzed the models for T1 in two different ways. First using all data available at T1 and second using only data of the completers, i.e., of those participants for whom data at both waves were available. Using all available data analysis allowed us to determine associations of correlates with high statistical power (due to the large sample size), while using completer data only lead to results which could be more easily compared between T1 and T2, as they contained the same subjects. To assess relative and absolute level stabilities we computed serial correlations and changes in mean values, respectively, between the two waves. To estimate effect sizes (Cohen’s d) for changes in mean values between waves, we used the method as described in Feingold (see Feingold, 2009; see the term *d*_GMA-RAW_ therein). Regarding regression model assumptions, multicollinearity was no issue with variance inflation factors (VIF) never exceeding the value 1.47 for any of the estimated models (see Supplementary material for more detailed information). Visual inspection of the distribution of the residuals using Q-Q plots and frequency distributions revealed no major deviation from normality. Each model was analyzed with and without potential outliers (based on Cook’s distance). Since results were comparable between the two variants, we only report here results based on data not excluding outliers. Apriori power analyses revealed that a predictor which is entered in the multiple regression model containing already seven predictors/covariates (as in our model) should have an effect size beta of 0.17 (i.e., small to medium) in a sample of size 260 (as in wave T2), given *α* = 0.05 and 1–β =0.8 to reach significance.

**Table 2:** Multiple regression model with outcomes BD or MD and correlates ERD, ARS, and alexithymia.

Sample	Outcome	Correlate	b	SE	*t*	df	*p*	*β*
All data at T1	BSQ-8C	DERS-SF	0.334	0.037	9.15	559	<0.001	0.37
Completers at T1	BSQ-8C	DERS-SF	0.354	0.053	6.67	242	<0.001	0.40
Completers at T2	BSQ-8C	DERS-SF	0.185	0.054	3.41	241	<0.001	0.22
								
All data at T1	BSQ-8C	TAS-20	0.005	0.038	0.14	559	0.890	0.01
Completers at T1	BSQ-8C	TAS-20	0.041	0.057	0.72	242	0.472	0.04
Completers at T2	BSQ-8C	TAS-20	0.132	0.054	2.45	241	0.015	0.16
								
All data at T1	BSQ-8C	ARS-D	0.694	0.076	9.09	559	<0.001	0.28
Completers at T1	BSQ-8C	ARS-D	0.649	0.111	5.86	242	<0.001	0.27
Completers at T2	BSQ-8C	ARS-D	0.769	0.117	6.57	241	<0.001	0.32
								
All data at T1	MDDI	DERS-SF	0.043	0.018	2.48	559	0.014	0.13
Completers at T1	MDDI	DERS-SF	0.059	0.025	2.32	242	0.021	0.17
Completers at T2	MDDI	DERS-SF	0.066	0.026	2.57	241	0.011	0.20
								
All data at T1	MDDI	TAS-20	0.063	0.018	3.44	559	<0.001	0.18
Completers at T1	MDDI	TAS-20	0.039	0.027	1.43	242	0.155	0.11
Completers at T2	MDDI	TAS-20	0.028	0.026	1.09	241	0.277	0.09
								
All data at T1	MDDI	ARS-D	0.069	0.037	1.89	559	0.059	0.07
Completers at T1	MDDI	ARS-D	0.091	0.053	1.72	242	0.086	0.10
Completers at T2	MDDI	ARS-D	0.047	0.056	0.84	241	0.399	0.05

The table contains the total scores of the following questionnaires: BSQ = Body Shape Questionnaire (short form); MDDI = Muscle Dysmorphic Disorder Inventory; ARS-D = Appearance-related Rejection Sensitivity; DERS-SF = Difficulties in Emotion Regulation Scale (short form); TAS-20 = Toronto-Alexithymie-Skala-20, PHQ-4 = Patient Health Questionnaire for anxiety and depression; EDE-Q8 = Eating Disorder Questionnaire (short form). The terms b and *β* denote the unstandardized and standardized regression coefficient, respectively. Covariates (not shown) were age, sex, educational status, BMI and COVID-19 phase.

## Results

### Sample characteristics

Response rate until May 2023 was 64.1% (i.e., 388 participants could have reached T2 by then). Participants’ age ranged from 14.6 to 24.9 years at T1, and from 15.8 to 26.2 years at T2. According to BMI (Weir and Jan, 2023) 10% (*n* = 62) of participants were underweight (< 18.5), 74% (*n* = 445) had a healthy weight (from 18.5 to 24.9), 13% (*n* = 79) were overweight (from 25 to 29.9) and 3% (*n* = 18) were obese (≥ 30). Completion of the online questionnaire at T1 occurred for 217 (36%) participants while COVID-19 restrictions were in force and for 388 (64%) participants after the lifting of COVID-19 restrictions. At the time of T2 all remaining COVID-19 restrictions in Switzerland had been lifted. The average mental health impairment was moderate at both measurement points with average PHQ-4 total scores of 7.8 (SD = 2.6) at T1 and 7.5 (SD = 2.6) at T2. Regarding global eating disorder psychopathology, the sample showed an EDE-Q4 total score of 2.0 (SD = 1.7) at T1 and 1.6 (SD = 1.4) at T2, these values lying both below the suggested cut-off score of 3.88 (Machado et al., 2020). Sample characteristics are summarized in Table 1. For T2 we compared responders with non-responders regarding the sample characteristics in Table 1. Controlled for multiple testing, no significant differences occurred between these two groups.

### Outcome body dissatisfaction

#### Correlates of body dissatisfaction

At T1, BD was positively associated with the two correlates ERD and ARS, whether all available data or only completers were considered (see Table 2). Corresponding effect sizes (b) were medium for ARS and medium to large for ERD. The association between BD and the alexithymia was very small and not significant.

At T2, BD was also positively associated with the two correlates ERD and ARS, while effect sizes were medium for ARS and small to medium for ERD. In contrast to T1, the association between BD and alexithymia at T2 was not negligible, though of small effect size.

#### Correlates of muscle dysmorphia

At T1, MD was positively associated with ERD, whether all available data or only completers were considered (Table 2). The effect size for this association was small to medium. In contrast, only small associations were found between MD and ARS at T1. The association between MD and alexithymia was small to medium when considering all available data, but only small when considering completers only.

At T2, MD was positively associated with ERD, with a small to medium effect size. Very small associations were found between MD and ARS and for alexithymia at T2.

### Absolute and relative level stabilities between waves T1 and T2

**Figure 1:**
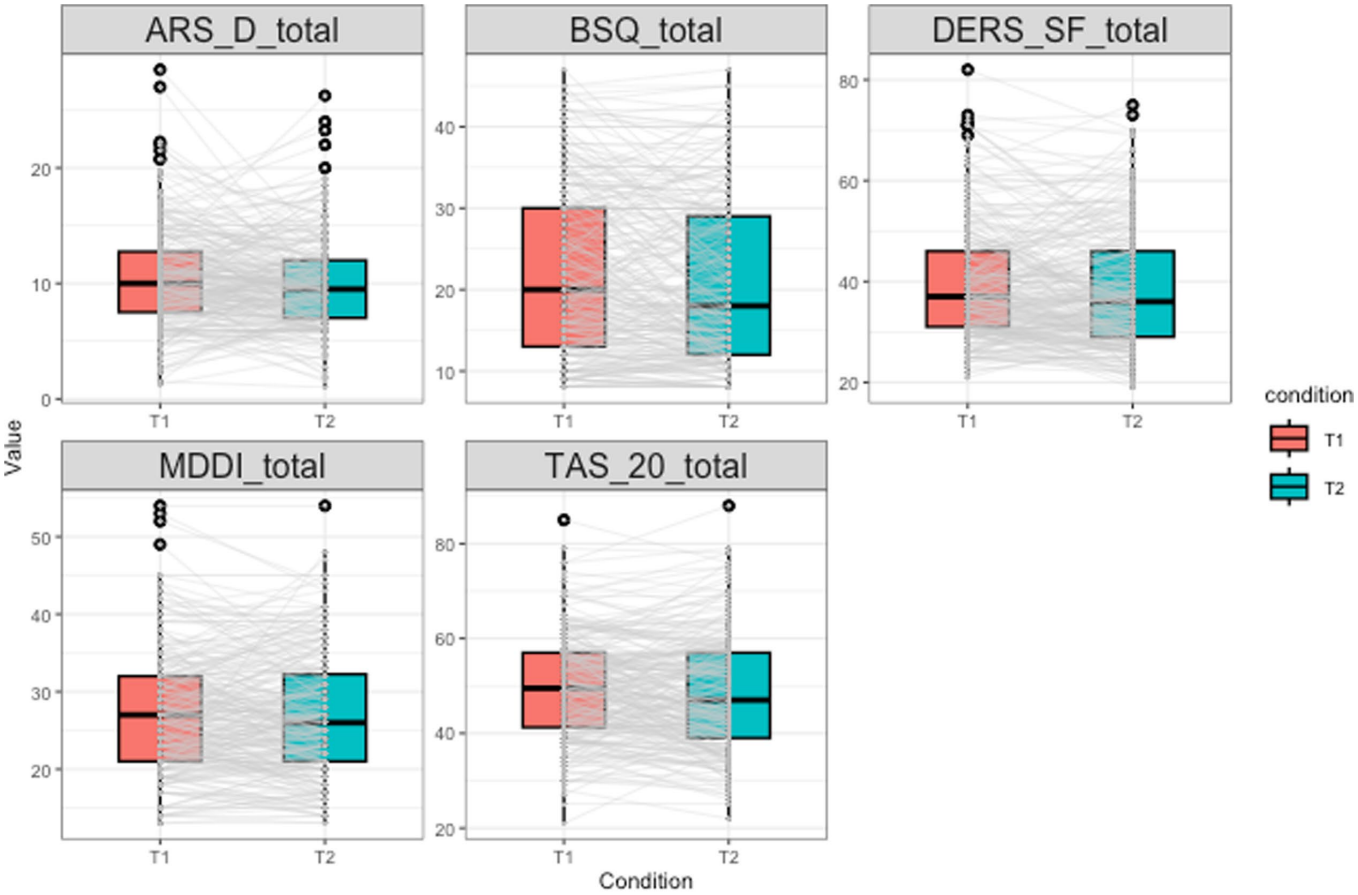
Boxplots showing the distribution of all five variables (correlates and outcomes) involved in the analyses at both waves. Gray lines connect the values of each individual participant between the two waves. This figure can be used to visually examine absolute level (boxplots) and relative level (grey lines) stability.

Relative level stabilities were in general high with serial correlations among the two waves varying between 0.63 and 0.75 (see Table 3; Figure 1) except for the ARS-D which had a considerably lower value of 0.45.

**Table 3:** Relative level stabilities of all 25 variables (correlates and outcomes) involved in the analyses.

Variable	*r*	*t*	*p*	*N*
BSQ-8C	0.749	17.8	<0.001	248
MDDI	0.704	15.6	<0.001	246
ARS-D	0.447	7.83	<0.001	246
DERS-SF	0.630	12.9	<0.001	252
TAS-20	0.661	13.8	<0.001	245

Serial correlations between waves T1 and T2. BSQ, Body Shape Questionnaire (short form); MDDI, Muscle Dysmorphic Disorder Inventory; ARS-D, Appearance-related Rejection Sensitivity; DERS-SF, Difficulties in Emotion Regulation Scale (short form); TAS-20, Toronto-Alexithymie-Skala-20; PHQ-4, Patient Health Questionnaire for anxiety and depression; EDE-Q8, Eating Disorder Questionnaire (short form).

Absolute level stabilities are summarized in Table 4 and Figure 1. Mean values of all variables hardly changed between waves. Strongest decreases were observed for BSQ-8C and TAS-20. Effect sizes for the change in the average value between T1 and T2 ranged between 0.03 and 0.14 and were thus all small to very small.

**Table 4:** Absolute level stabilities of all five variables (correlates and outcomes) involved in the analyses.

Variable	M (T1)	N (T1)	M (T2)	N (T2)	diff.	*t*	*p*	*d*
BSQ-8C	22.3	250	20.8	249	1.42	3.11	0.002	0.14
MDDI	27.1	248	26.9	247	0.23	0.59	0.559	0.03
ARS-D	10.5	248	9.99	247	0.47	1.65	0.100	0.11
DERS-SF	39.3	254	38.1	253	1.23	1.91	0.058	0.10
TAS-20	49.6	247	48.3	246	1.32	2.13	0.034	0.12

Relative level stabilities of all 5 variables (correlates and outcomes) involved in the analyses. Serial correlations between waves T1 and T2.

## Discussion

In the context of mental health in youth, BD has gained recognition as a significant public health concern (McLean et al., 2022) in females and males (Vuong et al., 2021), typically emerging during early adolescence (Rohde et al., 2015). For instance, BD has been identified as one of the most important and growing risk factors in the development and maintenance of eating disorders and also occurs in individuals with other mental disorders, as well as in the general population. Therefore, the main aim of the present study was to determine the association between different psychological factors on the development and persistence of BD.

### Psychological correlates of body dissatisfaction

On average, our participants showed mild symptoms of BD, despite the fact that the majority had a healthy weight, and relatively low eating disorder psychopathology at both waves (see Table 1). This is consistent with previous research, which indicates that BD is a prevalent and normative discontent in Western society populations, particularly during adolescence and young adulthood – a period characterized by heightened vulnerability in the onset and intensification of BD (e.g., Tantleff-Dunn et al., 2011; Rickwood, 2015; Wang et al., 2019). The results of our study are in line with other research conducted in the German-speaking region, which revealed that only 35% of females and 56% of males aged 13–17 were satisfied with their appearance (Widmer et al., 2018), while 40% of 16-20-year-old females and 18% of males reported BD (Narring et al., 2004).

Consistent with our hypothesis, there was a significant positive association between BD and the psychological factors of ERD and ARS at both waves. The effect sizes for ERD were medium to large at T1 and small to medium at T2, whereas the effect sizes for ARS were medium at both waves. Results are consistent with previous research, thereby highlighting the relevance of ERD and ARS in the onset and maintenance of BD in adolescents and young adults (e.g., Sim and Zeman, 2005; Hansson et al., 2016; Gardner et al., 2021; Morin and Meilleur, 2023). Moreover, our findings add to previous research underlining that the tendency to hastily feel rejected by the own appearance (ARS) may heighten the vulnerability to develop and maintain BD in youth (e.g., Park et al., 2009; Calogero et al., 2010; Schmidt and Martin, 2019).

Our study was the first to examine the association between alexithymia (inability to identify, understand, and express one’s own emotions, as well as a limited capacity to recognize and interpret emotions in others; Sifneos, 1973) and BD at two different data assessment waves one year apart. We found only very small associations between BD and alexithymia at T1, indicating no clinical importance. Furthermore, the association between BD and alexithymia at T2 was of small effect size, despite being significantly different from 0. In other words, if corroborated in future studies, it can be assumed that low levels of alexithymia, as measured in our study (indicated in Table 1) might not be important for the development of BD, whereas the observed moderate levels of ERD are important.

As mentioned above, the strength of these associations differed somewhat between the two waves. The discrepancies could be interpreted in two ways. First, they may point to developmental changes occurring over the course of the one-year study period. As previous studies have shown, adolescence and young adulthood are both characterized as periods with major physical, social, cognitive and emotional changes, and identity formation occurring (Rickwood, 2015; Voelker et al., 2015). According to a prior study (Steinberg, 2005) adolescence is characterized by an increased need to regulate affect and behavior in accordance with long-term goals and consequences. Due to the differential maturation – which can extend into young adulthood – of the developing brain, emotion, cognition, and behavior, this developmental phase is often characterized by heightened vulnerability (e.g., for the onset of mental health impairments) and adjustment. Second, these discrepancies might be a methodological issue as the sample size was much smaller in T2 than T1. However, since the completer sample at T1 revealed similar results compared to the full sample at T1, this suggests that developmental changes may have actually played a role in this context, though of limited size.

### Psychological correlates of muscle dysmorphia

Currently, there is limited knowledge regarding the mechanisms associated with MD, as the most frequently used BD questionnaires neglect to address dissatisfaction with and the drive for muscularity. Nevertheless, recent studies suggest a potential rise in the drive for muscularity and associated behaviors among males and females, due to cultural shifts in Western societies toward leaner, toned, and more muscular female body ideals (e.g., Girard et al., 2018; Cunningham et al., 2019; Mitchison et al., 2022). This highlights the importance of including measures that capture weight, shape, and muscularity concerns as significant aspects of BD in future mixed-gender cross- and longitudinal research. The present study is one of the first to examine MD in a general youth population, whereas previous research has primarily focused on individuals seeking treatment (e.g., body dysmorphic disorder) or those involved in bodybuilding or competitive sports. Our aim was to assess the impact of the psychological factors ERD, alexithymia, and ARS on MD symptoms. On average, the sample showed low MD symptoms at both waves according to the cut-off values reported elsewhere (Zeeck et al., 2018). It should be noted that our sample primarily consisted of young females (80% at T1, 84% at T2), which may have influenced the manifestation of MD in this study. Accordingly, research has shown that females are more likely to engage in strategies to lose or control weight, while males are more inclined to perform muscularity-enhancing strategies (Wyssen et al., 2016; Nagata et al., 2020; McLean et al., 2022).

In line with our hypotheses, the results revealed a significant positive association between MD and ERD at both waves, albeit with effect sizes being rather small. Note in this context that we made no attempt to control for multiple testing, and that doing so may have led to a non-significant result for this particular association (the corresponding *p*-values ranged between 0.11 and 0.21, see Table 2). Our study findings nevertheless contribute to the few studies which have included muscularity concerns in their analyses and demonstrated that higher levels of ERD are associated with increased levels of BD as well as engagement in muscle-building behaviors at both waves (Morin and Meilleur, 2023). Moreover, other research demonstrated that ERD amplify the association between BD and the desire for muscularity (Dakanalis et al., 2015; Cunningham et al., 2020). The limited influence of ERD on symptoms of MD in our study might be due to differences in sampling strategy. While other studies (e.g., Schmidt and Martin, 2019) mainly focused on individuals known for their vulnerability in MD (e.g., individuals seeking treatment for body dysmorphia or those involved in bodybuilding and competitive sport), we recruit youth from the general population. Hence, findings from prior research may be biased, resulting in a potential underestimation of MD and small effect sizes in our study, while also reducing comparability. Effect sizes for associations between MD and alexithymia and between MD and ARS were all small at best, except for the association between MD and alexithymia at T1 when considering all available data, which was highly significant, with small to medium effect size. At this point, it should be noted that there is scarce literature available in youth regarding this association, which complicates the interpretation and comparability of our data.

### Absolute and relative level stabilities between waves T1 and T2

We showed that both relative and absolute level stabilities were generally high in our sample. A high *relative level stability* means in the context of our study that participants with high/low values of psychological correlates of BD and of high values of BD and MD at T1, were likely to have corresponding high/low values at T2 one year later. This was, however, somewhat less the case for the ARS, where relative level stability was considerably lower compared to ERD, alexithymia, BD, and MD (see Table 3). This points to stronger individual fluctuations over the one-year period for ARS, in spite of the fact that the average value of ARS hardly changed within that period (absolute level stability, see Table 4). One explanation might be, that ARS often occurs within the context of peer victimization (e.g., Lavell et al., 2014; Schmidt and Martin, 2019), which we did not include in this study. Moreover, the rates of bullying may have been mitigated due to the COVID-19 pandemic and the accompanying restrictions (e.g., homeschooling). Similarly, a study involving Canadian students in grades 4–12 (*n* = 6,578; mean age 13.05 ± 2.34 years) provided further evidence, indicating significantly higher rates of bullying involvement before, compared to during the COVID-19 pandemic across all forms of bullying (general, physical, verbal, and social), except for cyberbullying, where the differences in rates were less pronounced (Vaillancourt et al., 2021). Thus, individual fluctuations could potentially be explained by varying levels of peer victimization in youth as well as lower rates of bullying during the COVID-19 pandemic. *Absolute level stabilities* were also high. Mean values of all investigated variables hardly changed between the two waves. Strongest changes were observed for BD and alexithymia, albeit with small effect sizes. In other words, in this study sample consisting of Swiss youth with predominantly high school or university educational background, the measured facets of ERD and subjective appearance-related rejection (ARS) experiences were of relevance to determine weight and shape concerns or concerns over being not muscular enough at two different time points, one year apart.

### Limitations and recommendations for future studies

The strengths of this study include the use of a relatively large sample of 605 participants at T1, the repeated assessment one year later (T2), and the use of online versions of short-form questionnaires that reduced participation burden and led to a comparably low drop-out rate. Another strength is, that we decided to also include concerns related to muscularity in our definition of BD, as increased desire for muscularity is detected in both, females and males (e.g., Phillips, 2011; Klimek et al., 2018; Cunningham et al., 2019; Nagata et al., 2020).

When interpreting the findings of the current study, several limitations have to be considered. In order to obtain more generally interpretable results, it would be beneficial to replicate our study in a more diverse sample with respect to educational status, as most of the participants were university (48%) or high school students (46%). Moreover, males were underrepresented with a participation rate of 19% at T1 and 14% at T2, which was already observed in previous mental health research studies (e.g., Ellis et al., 2014; Ryan et al., 2019), even though the current study made strong efforts to recruit males as well as participants from various educational levels via social media ads and reduced burden (e.g., online registration, short-form questionnaires). Therefore, in our study, we cannot draw meaningful conclusions about gender differences but instead controlled for the influence of gender. Given the data, since there are indeed gender differences with respect to the relevance of the muscularity, future studies should focus more specifically on this research question (Vuong et al., 2021). Findings on diverse study groups in terms of educational status and gender are important as they allow to better ensure that these groups also benefit from mental health research and that prevention as well as psychotherapy are tailored to their needs.

Moreover, our study findings cannot be generalized to the entire Swiss youth population, as the online-questionnaire study solely involved adolescents and young adults from the German-speaking region. As previous Swiss studies (e.g., Widmer et al., 2018) have identified differences in BD between Swiss language regions, future investigations should also include French- and Italian-speaking youth. It should further be noted that we shortened the Appearance-Based Rejection Sensitivity Scale (ARS-D; Schmidt and Martin, 2017), leading to reduced internal consistency and lower reliability values compared to those denoted in the literature (see assessment instruments; Park, 2007; Schmidt and Martin, 2017). There are also only few comparative studies on ARS in the German-speaking region due to the lack of assessment instruments. So far, only the ARS-D (original version: Park, 2007) is available, which was translated and validated by Schmidt and Martin (2017), mostly for well-educated females aged 18–55 years (*n* = 391; 74.9% female; 47.8% high school; 26.9% university; Schmidt and Martin, 2017). This is why we had to make linguistic and content adjustments for our age group (e.g., scenarios were missing where youth experienced appearance-based rejection on social media and/or peer victimization). In the future, studies are needed to adapt and validate ARS questionnaires for adolescents and young adults. In addition, it must be noted that BD/MD and ARS in other studies were assessed using many different questionnaires, which complicates the comparability of the findings. Another limitation of this study is that we did not differentiate between diverse sexual orientation categories (e.g., hetero-, homo-, bisexual). Although, evidence has shown that especially homosexual male adolescents and young adults, tend to exhibit higher levels of BD and display a greater drive for thinness and muscularity (Cella et al., 2010; Jankowski et al., 2014). It is therefore possible that psychological correlates of BD/MD may have a different impact depending on sexual orientation, which is why sexual orientation should be considered as an additional variable in future research. At this point, it should also be noted that there is currently no validated German version of the DERS-SF available. Nevertheless, in our study sample, Cronbach’s alpha and McDonald’s omega were 0.90 and 0.93, respectively, thus providing evidence that the German version of the DERS-SF can be effectively used in adolescents and young adults.

Furthermore, sociocultural factors, such as the media-promoted thin- and muscularity ideals and the associated degree of internalization of these ideals as well as appearance pressure from peers and families to conform to these ideals have been shown to highly impact youths’ BD and could be included in future research (e.g., Thompson et al., 1999; Ricciardelli and Yager, 2015). Given that exposure to these appearance ideals is frequent, especially through image-based social media platforms (e.g., Instagram, TikTok) and advertisements, future research should prioritize investigating cognitive processes, such as thought-shape fusion (e.g., Wyssen et al., 2016; Munsch et al., 2021a,b). Additionally, there should be a particular focus on exploring resulting unhealthy body change behaviors, such as weight reduction or muscle gain strategies (Neumark-Sztainer et al., 2006; McLean et al., 2022).

### Conclusion and practical implications

The findings of this study underline the punctual and potentially longer lasting cross-sectional associations between ERD, alexithymia and ARS in a youth sample and concerns about their body shape, weight and muscularity at two assessment time points one year apart. They need to be corroborated but highlight the pertinence to develop and validate prevention and treatment modules to address emotion regulation competences and coping with subjective appearance-related rejection sensitivity already at a young age. Future studies including assessment of these mechanisms in daily life and the evaluation of the effect of corresponding treatment modules will help to enhance our understanding of the role and stability of these processes (i.e., Munsch et al., 2021a,b).

## Data availability statement

The data analyzed in this study is subject to the following licenses/restrictions: as the online questionnaire study is still running, we have not yet published the data sets. Requests to access these datasets should be directed to SM, simone.munsch@unifr.ch.

## Ethics statement

The studies involving humans were approved by Cantonal ethical committee of Bern, Switzerland. The studies were conducted in accordance with the local legislation and institutional requirements. The ethics committee/institutional review board waived the requirement of written informed consent for participation from the participants or the participants’ legal guardians/next of kin because no risks or disadvantages were associated with participation.

## Author contributions

VM: Writing – original draft. FF: Writing – review & editing. AM: Data curation, Writing – review & editing. SM: Writing – review & editing.
