# Supplementary material for: Psychological correlates of body dissatisfaction in Swiss youth over a one-year study-period
Source: Front Psychol. 2024 Jan 8;14:1269364. doi: 10.3389/fpsyg.2023.1269364 (PMC10802120; doi:10.3389/fpsyg.2023.1269364)
Supplement: Supplementary file 1 [file Data_Sheet_1.pdf]

Supplementary Material

TABLE 1 | Correlation matrix of for correlates and covariates included in the regression models (n=572).

	Age	Sex	Education	BMI	COVID-19	DERS-SF	ARS-D
Age	–						
Sex	–0.03	–					
Education	–0.04	0.09	–				
BMI	0.18	0.04	–0.09	–			
COVID-19	–0.26	0.02	–0.08	0.07	–		
DERS-SF	–0.06	–0.12	–0.03	0.16	0.13	–	
ARS-D	–0.05	–0.16	–0.06	0.10	0.08	0.21	–
TAS-20	–0.26	–0.04	–0.07	0.05	0.22	0.64	0.22

TABLE 2 | Correlation matrix for regression model with all available cases at wave 1.

	VIF scores
Age	1.22
Gender	1.12
Education	1.06
BMI	1.11
COVID-19	1.13
DERS-SF	1.88
ARS-D	1.10
TAS-20	1.93
